# The Role of Extracellular Adenosine Generation in the Development of Autoimmune Diseases

**DOI:** 10.1155/2018/7019398

**Published:** 2018-03-26

**Authors:** F. Morandi, A. L. Horenstein, R. Rizzo, F. Malavasi

**Affiliations:** ^1^Stem Cell Laboratory and Cell Therapy Center, Istituto Giannina Gaslini, 16148 Genova, Italy; ^2^Department of Medical Sciences, Laboratory of Immunogenetics, University of Torino, 10126 Torino, Italy; ^3^CeRMS, University of Torino, 10126 Torino, Italy; ^4^Department of Medical Sciences, Section of Microbiology, University of Ferrara, 44121 Ferrara, Italy

## Abstract

Adenosine (ADO) is an immunosuppressive molecule, which suppresses the immune responses by interacting with specific receptors expressed by immune effector cells. ADO is produced from ATP through the enzymatic activities of CD39 and CD73. Alternatively, ADO can be generated starting from NAD^+^, which is metabolized by the concerted action of CD38, CD203a/PC-1, and CD73. The role of ADO in immunity has been characterized in the last years in physiology and in pathological settings. This review examines a panel of reports focused on the functions of ADO in the context of human autoimmune/inflammatory diseases and the selected animal models. The final aim is to consider the role of adenosinergic ectoenzymes and ADO receptors as novel therapeutic targets for selected diseases.

## 1. Ectonucleotidases and Adenosine

Adenosine (ADO) is a nucleoside with pleiotropic functions, which acts as an intracellular and extracellular mediator of multiple biological processes. The concentration of ADO in the interstitial fluids, which is generally low, dramatically increases during tissue injury, caused by hypoxia, ischemia, inflammation, and cancer. ADO acts as a danger signal, by activating specific ADO receptors (ADOR), namely, A1, A2A, A2B, and A3, different in function and tissue distribution [1].

A1 and A3 receptors are coupled with G proteins of the Gi, Gq, and Go family, and their activation leads to the release of calcium ions from intracellular stores. In contrast, A2A and A2B receptors are coupled with G proteins Gs or Gq and activate adenylyl cyclase or phospholipase C. Moreover, all adenosine receptors activate mitogen-activated protein kinase (MAPK) pathways, which include extracellular signal regulated kinase 1 (ERK1), ERK2, Jun N-terminal kinase, and p38 MAPK. ADO also has receptor-independent effects, because extracellular adenosine can cross the cell membrane and activate AMP-activated protein kinase (AMPK), adenosine kinase, and S-adenosyl homocysteine hydrolase pathways [2].

Upon interaction with these receptors, ADO can trigger different cellular responses, aimed at restoring tissue homeostasis. Among them, ADO can limit inflammatory and immune responses, to avoid tissue damage and promote the healing process [2]. Indeed, ADO acts as an immunosuppressive molecule, able to inhibit the functions of different cell populations and subsets of the immune system, including T and B lymphocytes, NK cells, dendritic cells, monocytes, and macrophages [3–6].

ADO is produced through the action of adenosinergic ectoenzymes expressed on the membrane of different cell populations. ADO may be obtained by metabolizing ATP (canonical pathway) or NAD^+^ (alternative pathway). The canonical pathway is started by CD39, an ectonucleoside triphosphate diphosphohydrolase (NTPDase), which converts ATP to ADP. CD39 can also convert the latter molecule into AMP, fully dephosphorylated to ADO by the 5′-nucleotidase (5′-NT) CD73 [7]. CD39 and CD73 have been recently proposed as novel checkpoint inhibitor targets, since ADO generated by these ectonucleotidases interferes with antitumor immune responses [8].

We have recently demonstrated that ADO can also be generated from the NAD^+^ substrate through an alternative pathway, where CD38 (a NADase and ADP-ribosyl cyclase) plays a central role. CD38 converts NAD^+^ to ADPR, which in turn is metabolized to AMP by CD203a/PC-1 (an ectonucleotide pyrophosphatase phosphodiesterase 1). CD203a/PC-1 can also convert ATP to AMP, which is eventually degraded to ADO by CD73, a molecule that is shared between the two pathways [9, 10].

ADO levels can be regulated by intracellular and extracellular mechanisms, through the action of (i) nucleoside transporters, namely, equilibrative nucleoside transporters (ENT1, ENT2, ENT3, and ENT4) and concentrative nucleoside transporters (CNT1, CNT2, and CNT3), that are able to transport ADO inside the cells [2, 11] and (ii) adenosine deaminase (ADA1 and ADA2), which is expressed by different cell populations and is able to convert ADO into inosine [12, 13]. However, inosine can also induce immunosuppressive effects, through the interaction with the A2a receptor [14].

### 1.1. Regulatory Cells with Adenosinergic Ectoenzyme Expression

Adenosinergic ectoenzymes are present on the surface of different regulatory cell populations. CD4^+^CD25^high^FOXP3^+^ regulatory T cells (Tregs) express high levels of CD39 and CD73. The ADO produced is believed to be instrumental in abrogating the effector T cell functions after interacting with ADORA2A. The inhibitory effect can be counteracted by effector T lymphocytes through the activity of ADO deaminase (ADA). ADA, which is responsible for adenosine degradation, is hosted on CD26, a cell surface-bound glycoprotein [15]. Also, CD56^bright^CD16^−^ NK cells play multiple roles in the regulation of immune response. We recently demonstrated that CD56^bright^CD16^−^ NK cells express high levels of CD39, CD73, CD203a/PC-1, and CD157, as compared with the CD56^dim^CD16^+^ NK subset. Moreover, CD56^bright^CD16^−^ NK cells produce ADO and have the ability to inhibit autologous CD4^+^ T cell proliferation. CD38 has a central role in this process [16].

Another important regulatory cell subset is represented by CD45R0^+^CD4^+^CD49b^+^LAG-3^+^ type 1 regulatory T cells (Tr1). CD39 was recently demonstrated as promoting Tr1 cell differentiation by depleting extracellular ATP [17]. More importantly, CD39 is expressed on Tr1 cells and contributes to their suppressive activity through the generation of ADO in a discontinuous fashion. In fact, CD73, the other component of the classical pathway for ADO production, is expressed by responder T cells rather than Tr1 cells [17].

Regulatory cells are also present among B lymphocytes. A B cell subpopulation, characterized as CD39^+^CD73^+^, generates ADO, and it is endowed with regulatory properties [18]. These cells are expanded *in vitro* through ADORA1- and A2A-mediated autocrine signaling and are able to inhibit effector T cell functions through the production of ADO and IL-10 [19].

Myeloid-derived suppressor cells (MDSC) and mesenchymal stem cells (MSC) are populations with regulatory functions, characterized by the presence of adenosinergic ectoenzymes. A recent report indicates that CD11b^+^CD33^+^ MDSC in peripheral blood and tumor tissues from nonsmall cell lung cancer (NSCLC) patients express surface ectonucleotidases CD39 and CD73. Such expression is related to the immunosuppressive and chemoprotective effects of MDSC, which favor NSCLC progression [20]. On our side, we showed that MDSC purified from the bone marrow (BM) niche of multiple myeloma patients express CD39 and CD73, which contribute to the local production of ADO. ADO in the BM is expected to lead to an impairment of the antitumor immune response [21]. The same study pointed out that MSC also express CD38, CD157, and CD203a/PC-1 [21]. Other reports confirmed the presence of CD39 and CD73 in MSC. These cells may lead to the generation of ADO when ATP concentrations are high, such as tissue injuries. However, this is achieved only with the cooperation of activated T lymphocytes, which express high levels of CD39 [22]. On the other hand, other reports demonstrated that cancer-derived MSC express functional CD39 and CD73, per se able to produce ADO and hence to suppress T cell functions [23].

**Figure 1:**
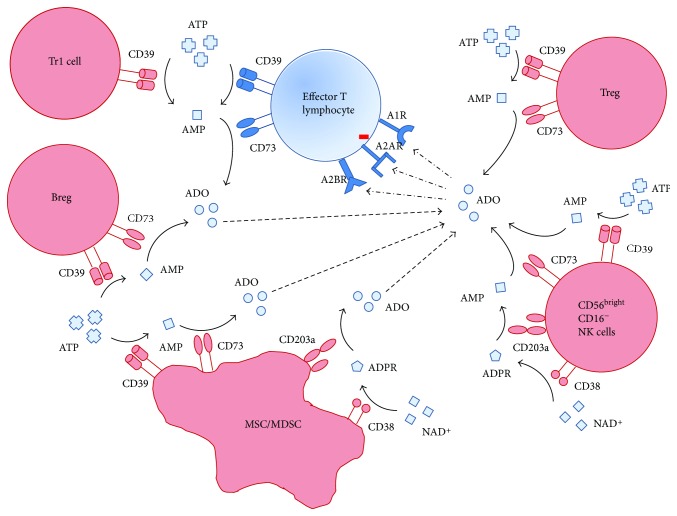
Schematic representation of (i) the expression of adenosinergic ectoenzymes on different regulatory cell subsets, (ii) their function in the generation of ADO, and (iii) the inhibitory effect of ADO on immune effector cells.

A schematic representation of the expression and function of adenosinergic ectoenzymes on regulatory cell populations is shown in Figure 1.

## 2. Autoimmune Diseases

Autoimmune diseases are chronic conditions initiated by the loss of immunological tolerance to self-antigens and represent a heterogeneous group of disorders that afflict specific target organs or multiple organ systems. The frequent autoimmune diseases include RA (rheumatoid arthritis), SLE (systemic lupus erythematosus), MS (multiple sclerosis), T1DM (type I diabetes mellitus), JIA (juvenile idiopathic arthritis), AIH (autoimmune hepatitis), and so on. The basic function of the human immune system is to distinguish antigens and then eliminate the non-self antigens, to protect the body from infection, tumor, and so on. In normal situations, there is an “alarm” signal which generates various cellular responses that aim to prevent excessive inflammatory response and restore the immune homeostasis. The possible implication of a common point between autoimmune diseases is supported by the presence of common phenotypes between autoimmune disorders: (i) signs and symptoms such as arthralgia, arthritis, alopecia, fatigue, photosensitivity, and Raynaud's phenomenon; (ii) nonspecific autoantibodies (i.e., antinuclear antibodies, rheumatoid factor, and anti-Ro antibodies); (iii) high levels of cytokines (i.e., TNF, IL-1, IL-6, IL-10, IL-17, etc.); and (iv) presence of infiltrating cells as phagocytic macrophages, neutrophils, self-reactive CD4^+^ T helper cells, and self-reactive CD8^+^ cytolytic T cells along with smaller numbers of natural killer cells, mast cells, and dendritic cells. Among the T effector cells, Th1, Th17, and Th9 cells contribute to the pathogenesis of autoimmune diseases [24]. In this review, we will point out the implication of ADO in five autoimmune diseases and/or their corresponding animal models: rheumatoid arthritis, multiple sclerosis, juvenile rheumatoid arthritis, autoimmune uveitis, and diabetes mellitus.

Rheumatoid arthritis (RA) is an autoimmune disorder primarily affecting the joints. The underlying mechanism involves a combination of genetic and environmental factors. Several cytokines and their receptors are overexpressed in the synovium, and among them, TNF-*α* seems to play an important role, and it has been proposed as a therapeutic target [25]. In fact, RA patients treated with the anti-TNF-*α* antibody (infliximab), alone or in combination with methotrexate (MTX), showed clinical benefits [26].

Multiple sclerosis (MS) is the most famous autoimmune disease attacking the central nervous system. The physiology of MS is monitored by activation of immune-inflammatory, oxidative, and nitrosative stress pathways, related with two main steps: (i) myelin sheath destruction and formation of lesions and (ii) inflammation [27].

Juvenile rheumatoid arthritis is the most common chronic rheumatic disease of childhood and a leading cause of short- and long-term disability. The synovial lymphocyte infiltrate consists mainly of Th1 T cells and, like rheumatoid arthritis, is believed to be a polarization towards Th1 proinflammatory response [28].

Autoimmune uveitis, an inflammatory noninfectious process of the vascular layer of the eye, can lead to visual impairment and, in the absence of a timely diagnosis and suitable therapy, can even result in total blindness. T cell effector phenotypes and their cytokine pathways seem to be implicated into autoimmune uveitis: T helper 1 (producing interleukin-2 and interferon-*γ*) and T helper 2 cells (producing interleukins 4, 5, and 13) exert both pathogenic and protective roles, whereas T helper 9 (interleukins 9 and 10) and T helper 17 cells (interleukins 17A, 21, and 22) play exclusively pathogenic roles [29]. Recently, T helper 17 cells have been also implicated in the uveal pathology [30].

Type 1 and 2 diabetes are both caused by immune-mediated destruction and dysfunction of pancreatic beta cells, eventually causing defective glucose-stimulated insulin secretion (GSIS) and beta cell apoptosis in diabetes. Recent findings indicate that inflammation and autoimmune recognition of beta cells are triggered by a local production of diabetogenic cytokines, such as IFN-*γ*, IL-1*β*, and TNF-*α*, by infiltrated immune cells in pancreatic islets [31, 32].

## 3. Adenosine and Human Autoimmune Diseases

Several lines of evidence have demonstrated that ADO behaves as the “alarm” signal *in vivo*. Adenosine activates ADO receptors on target cells, generates multiple cellular responses, and then suppresses the immune response [33]. It has been demonstrated that locally produced ADO can stimulate the release of anti-inflammatory cytokines (i.e., IL-10) and inhibit the release of proinflammatory molecules (i.e., TNF-*α* and nitric oxide) in animal models of inflammation [34]. Indeed, multiple reports suggest that the onset of autoimmune disorders is—at least in part—related to a partial or complete loss of function of the adenosinergic pathways and to a local defective production of ADO.  Table 1 summarizes all these findings.

### 3.1. Adenosine and Multiple Sclerosis

Notions accumulated in recent years indicate the existence of a link between ADO production and clinical outcome of patients with multiple sclerosis (MS). Airas et al. demonstrated that endothelial cells (ECs) treated with IFN-*β* are able to increase CD73 surface expression *in vitro*. The same effect was also observed on ECs after systemic treatment of MS patients *in vivo* with IFN-*β*, where an increase in soluble serum CD73 was also observed. Moreover, a correlation between increase in CD73 and clinical outcome of MS patients is reported [35]. In line with this observation, IFN-*β* was shown as increasing the expression of CD73 on blood-brain barrier- (BBB-) derived EC and also on astrocytes. This modulation was followed by a reduced transmigration of lymphocytes through BBB-EC. This specificity of action was confirmed by using *α*,*β*-methyleneadenosine-5′-diphosphate, a specific inhibitor of CD73, which restored the situation *quo ante*. At the same time, the same group of authors found that the IFN-*β* treatment was followed by increased levels of soluble serum CD73 activity and skin microvascular CD73 expression in the majority of MS patients. This study suggested that ADO might contribute to the beneficial effects of IFN-*β* observed on MS patients [36]. Fletcher et al. demonstrated that CD39, an ectonucleotidase which hydrolyzes ATP, may be also related to MS progression. Indeed, CD39 is expressed on a subset of Tregs. Both CD39^+^ and CD39^−^ Treg subsets are able to dampen IFN-*γ* production by effector T cells, whereas only CD39^+^ Tregs are able to suppress IL-17 production by Th17 cells. They found that frequency and suppressive functions of CD39^+^ Tregs are decreased in MS patients and that the inhibition of Th17 cells was related to ADO production through the enzymatic activity of CD39 and CD73 [37].

Other evidences were inferred by the results of studies in the field of experimental autoimmune encephalomyelitis (EAE), an animal model for MS. CD73 expression on Th17 cells appears as increased during disease progression. However, CD73 deficiency did not affect either EAE development or induction of IL-17, IFN-*γ*, or GM-CSF, Th17-associated cytokines. Another finding was that CD73 is not required to either promote or limit Th17 cell expansion *in vitro* [38]. In line with these findings, Millis et al. showed that cd73^−/−^ mice are resistant to EAE. However, CD4^+^ T lymphocytes from these mice produced levels of proinflammatory cytokines higher than those observed from wild-type (WT) mice. Furthermore, they may induce EAE when transferred in cd73^+/+^ T cell-deficient mice. They found that cd73^−/−^ mice display a lower number of infiltrating lymphocytes in their central nervous system (CNS) than do WT mice. Since they observed a lack of CD73 expression on brain endothelial cells, they concluded that CD73 expression and ADO production are required for the recruitment of lymphocytes into the CNS during EAE development [39]. Other reports showed that such effects were achieved through the induction of CX3CL1 as expressed at the choroid plexus. Indeed, CX3CL1 was upregulated in the brain at the acute stage of EAE in WT mice, but not in CD73 KO animals. Moreover, blockade of ADORA2A following EAE induction prevented disease development and the induction of brain CX3CL1 expression. Another finding was that CX3CL1 expression can be upregulated in the brain by antagonizing ADORA2A action. Finally, CX3CL1 blockade protected from EAE development and inhibited lymphocyte entry into the CNS [40]. Hydrolysis of ATP, ADP, and AMP in EAE mice is decreased in blood serum and increased in spinal cord membrane preparations as compared to control mice. A reasonable interpretation is that the function of CD39 and CD73 is regulated during the progression of the disease. An extension of this view is that these molecules represent a target for novel therapeutic strategies in immune-mediated diseases, primarily in MS [41].

Another study demonstrated that ADORA2a was upregulated on T cells and macrophages/microglia in the CNS of EAE mice. Accordingly, ADORA2a-deficient mice showed an exacerbated disease, with increased frequencies of IFN-*γ*-, IL-17-, and GM-CSF-producing CD4^+^ T cells and higher numbers of inflammatory lesions in the early stage. However, EAE quickly ameliorated and myelin debris accumulation was lower in ADORA2a^−/−^ mice. Finally, activation of ADORA2a inhibited myelin phagocytosis by macrophages/microglia *in vitro* as well as migration of T cells, macrophages, and primary microglia [42].

ADORA1 is also involved in the development of EAE. Indeed, it has been demonstrated that ADORA1^−/−^ mice developed a severe progressive-relapsing form of EAE, as compared to WT mice, with worsened demyelination, axonal injury, and enhanced activation of microglia/macrophages. In addition, proinflammatory gene expression was upregulated, whereas anti-inflammatory genes were suppressed [43].

### 3.2. Adenosine and Rheumatoid Arthritis

Several authors have demonstrated the role of ADO and adenosinergic ectoenzymes in the context of RA. Flogel et al. demonstrated that the expression of CD73 and ADORA2A is strongly upregulated on neutrophils and inflammatory monocytes isolated from synovial fluids of mice with collagen-induced arthritis (CIA), a well-established mouse model of RA, as compared to controls [44]. Another study was focused on the analysis of the susceptibility of CD73^−/−^ mice to CIA. CD73-deficient mice are significantly more susceptible to CIA than are wild-type mice. Further, the production of proinflammatory cytokines in the joints, Th1 T cell responses, and joint destruction were all increased. Moreover, a delayed anticollagen IgG response was detected in CD73-deficient mice, thus suggesting a defective isotype class switching. The authors have demonstrated that CD73 expression on nonhematopoietic cells was important for protecting from CIA development [45]. CD39^+^ Tregs have been characterized also in the context of RA. Indeed, it has been demonstrated that the frequency of this cell subset is increased in the peripheral blood (PB) of RA patients and that these cells are also abundant in the synovial fluid (SF). Also in the synovium, CD39^+^ Tregs are able to suppress different effector T cell functions, including production of IFN-*γ*, TNF, and IL-17F, but they do not limit IL-17A secretion, as previously reported [46]. The role of CD39^+^ Tregs as key factors as immune modulators in the context of RA has been demonstrated by Peres et al., who have analyzed the frequency of this cell subset in the PB of RA patients treated for three months with MTX. They found that such frequency was increased in RA patients that were responsive to MTX, but not in unresponsive patients, as compared to healthy controls. Furthermore, they demonstrated that Tregs from unresponsive patients express lower levels of CD39, produce less amounts of ADO (starting from ATP), and display a decreased regulatory activity, as compared to Tregs from MTX-responsive patients. In a prospective study, they demonstrated that unresponsive patients displayed a lower expression of CD39 on Tregs than did responsive patients and healthy controls. Moreover, CD39 blockade dampened the antiarthritic effect of MTX treatment in a murine model of RA, thus suggesting that CD39 and ADO production is involved in the mechanism of action of MTX and is required for the responsiveness to the treatment. The authors concluded that low expression of CD39 on Tregs could represent a biomarker for identifying MTX-resistant RA patients [47].

### 3.3. Adenosine and Juvenile Idiopathic Arthritis

We have demonstrated an altered function of adenosinergic ectoenzymes in the context of juvenile idiopathic arthritis (JIA), a pediatric form of RA. In particular, the expression and function of CD38 and CD73 were decreased in CD16^−^CD56^bright^ NK cells isolated from synovial fluid of JIA patients. These cells displayed an altered kinetic for ADO production and failed to inhibit autologous CD4^+^ T cell proliferation, as compared to those isolated from healthy controls [16]. Other authors have demonstrated that, in JIA patients, the expression of CD73 on CD19^+^ and CD8^+^ lymphocytes was lower in cells isolated from SF than in those from PB. Consequently, SF CD8^+^ cells produced less amounts of ADO than did PB counterparts. Finally, they found that CD73 expression in SF cells was lower in patients with an extended oligoarticular form than in those with milder forms, thus suggesting that CD73 expression and ADO production correlated with disease severity, due to a low anti-inflammatory activity [48].

### 3.4. Adenosine and Autoimmune Uveitis

A putative role of ADO has been also described in experimental autoimmune uveitis (EAU), a murine model of human autoimmune uveitis. It has been demonstrated that the recovery from EAU is mediated by suppressor antigen-presenting cells, which present autoantigens and produce ADO to activate antigen-specific Tregs, through ADORA2A. These inducible Tregs can, in turn, suppress the autoimmune disease through a PD-1/PD-L1-dependent mechanism [49]. It has been also demonstrated that mesenchymal stem cells (MSC) are able to attenuate the severity of the disease. Such therapeutic effect was reverted by pretreating MSC with a CD73 inhibitor, thus suggesting that this immune-modulatory activity was related to ADO, which is produced through the cooperation between CD73 and CD39 and in turn abrogates T cell functions [50]. *γδ* T cells can either enhance or inhibit an adaptive immune response and are important in the context of EAU. It has been reported that *γδ* T cells expressed CD73 during the different stages of EAU, and a low CD73 expression on *γδ* T cells correlated with enhanced Th17 response. The expression of CD73 on *γδ* T cells is transiently downregulated upon activation. Moreover, *γδ* T cells isolated from CD73^−/−^ mice are more potent at inducing a Th-17 response and eye pathology in *γδ*^−/−^-recipient mice than are *γδ* T cells isolated from WT mice. On the other hand, *γδ* T cells isolated from WT mice, but not those isolated from CD73^−/−^ mice, are able to inhibit T cell proliferation *in vitro*. Collectively, these data suggest that targeting CD73 expression on *γδ* T cells might control both their pro- or anti-inflammatory effects [51].

### 3.5. Adenosine and Diabetes

A possible role of adenosinergic ectoenzymes and ADO has been recently hypothesized in a multiple low-dose streptozotocin- (MLDS-) induced diabetes, a murine model of human T1DM. In particular, the authors investigated the role of CD39, using wild-type mice, CD39 KO mice, and mice overexpressing CD39 that were subsequently subjected to MLDS. They demonstrated that CD39KO mice developed diabetes more rapidly and with higher frequency than did WT mice. In contrast, overexpression of CD39 protected mice from diabetes, through the production of ADO and activation of ADORA2A and ADORA2B [52]. In this line, it has been previously demonstrated that mice lacking ADORA2A (A2AR KO) are highly susceptible to MLDS-induced diabetes, with the presence of hyperproliferative T cells [53]. In contrast, CD73 KO mice were protected from MLDS-induced diabetes as compared to wild-type mice [54].

## 4. Adenosine in Other Inflammatory Diseases

Inflammatory diseases include autoimmune diseases and a vast array of disorders and conditions that are characterized by inflammation. This includes allergy, asthma, celiac disease, glomerulonephritis and other renal diseases, hepatitis, inflammatory bowel disease, preperfusion injury, and inflammatory diseases related to transplant. We have here investigated the role of ADO in the context of skin allergy, renal inflammatory diseases, and graft-versus-host disease (GvHD).

### 4.1. CD73 in Inflamed Human Skin

CD73 is highly expressed on endothelial cells and plays a role in mediating the adhesion of leukocytes to endothelial cells. Consequently, CD73 regulates the entry of the latter cells in the vascular network and, in turn, their recruitment to the site of inflammation. Moreover, ADO produced by CD73^+^ endothelial cells exerts anti-inflammatory effects, helping to maintain the endothelial barrier function [55–57]. Arvilommi et al. have investigated the role of CD73 in the migration of activated lymphocytes to inflamed skin, in the context of idiopathic and allergic disorders of the skin. They demonstrated that CD73 is also expressed on activated lymphocytes, and skin-homing lymphocytes mostly expressed both CD73 and cutaneous lymphocyte antigen (CLA). Moreover, the treatment of peripheral blood lymphocytes with an anti-CD73 monoclonal antibody significantly reduced the binding of the latter cells to the vascular endothelium of the inflamed skin, thus suggesting the role of CD73 in the recruitment of activated lymphocytes in this context [58].

### 4.2. CD73 in Renal Diseases

The important role of CD73 on endothelial cells in the control of leukocyte trafficking has been investigated also in mice with renal injury. In fact, CD73^−/−^ mice showed autoimmune inflammation with glomerulitis and peritubular capillaritis, glomerular deposition of immunoglobulins, and complement and enhanced presence of lymphocytes and macrophages in the interstitium. Moreover, vascular inflammation was associated with enhanced serum levels of proinflammatory cytokines and chemokines. Finally, a reduced number of podocytes and endothelial fenestrations were observed in CD73^−/−^ mice. These data demonstrated that a lack of CD73 reduced renal function and is associated with autoimmune inflammation [59].

### 4.3. Adenosine and Graft-versus-Host Disease

MSC exert their regulatory activity through the release of soluble immunosuppressive molecules, such as TGF-*β*1, HGF, IDO, PGE_2_, IL-10, and HLA-G [60]. For their immunosuppressive potential, MSC have been proposed as a therapeutic tool in the treatment of severe acute GvHD. Recently, Sangiorgi et al. have demonstrated that, upon stimulation with a toll-like receptor 9 agonist, bone marrow- (BM-) derived MSC (i) upregulated CD73 and CD39 expression on their surface and (ii) showed increased immunosuppressive functions. Thus, they conclude that ADO, generated by the CD39/CD73 pathway, may also play a role in the immunomodulatory function of MSC [61].

Like MSC, human-derived gingival mesenchymal stem cells (GMSCs) are able to suppress immune responses and T cell-mediated CIA in animals. It has been demonstrated that GMSCs are able to suppress the proliferation of PBMC and T cells *in vitro*. When cotransferred with PBMC in a murine model of GvDH, GMSCs are able to suppress the engraftment of human cells and to prolong the survival of mice. It has been demonstrated that the mechanism of GMSC-mediated immunosuppression involves the enzymatic function of CD39 and CD73 and production of ADO. These cells represent a potential clinical option for the treatment of GvHD and autoimmune diseases [62].

The role of CD73 and ADO in the control of GvHD was also supported by the finding that Tregs from CD73 KO mice are unable to mitigate GvHD mortality, compared to WT Tregs. Moreover, blockade of the ADO A2a receptor exacerbated GvHD, thus suggesting that ADO is involved in this process, through the interaction with the A2a receptor. Finally, blockade of CD73 induced the expansion of alloreactive T cells, which exacerbated GvHD and enhanced the graft-versus-tumor effect. Collectively, these data demonstrated that blocking CD73 activity might be relevant in the context of BM transplantation [63, 64].

## 5. Therapeutic Implications for Autoimmune Disorders

Several studies support the concept that ADO, as well as enzymes belonging to adenosinergic pathways for ADO production, might represent promising therapeutic targets for autoimmune and inflammatory diseases.

Flogel et al. analyzed the effects of 2-(cyclohexyl methylthio) adenosine 5′-monophosphate (chet-AMP), a phosphorylated ADORA2A agonist (prodrug) that requires CD73 activity to become activated, in mice with CIA. The administration of chet-AMP triggers immunosuppressive and anti-inflammatory activities, through a CD73-mediated conversion of chet-AMP to functional chet-ADO. These findings suggested that ADOR agonists may represent a novel therapeutic target for patients with RA [44]. Similar data were obtained in another study, where the administration of an ADORA2A agonist to CD73^−/−^ mice resulted in arthritis incidence similar to wild-type mice, thus confirming the protective role for A2A signaling in the context of CIA [45]. Similar results have been obtained using agonists of ADORA3: the mechanism underlying this beneficial anti-inflammatory effect was related to the suppression of TNF-*α* production [65, 66].

Ingwersen et al. described a dual role for ADORA2a in the context of EAE. Indeed, preventive treatment with the ADORA2a-specific agonist ameliorated disease, whereas treatment with the same agonist after disease onset exacerbated disease progression and tissue destruction. These data suggested that ADORA2a provides anti-inflammatory effects on T cells and protection at early stages, whereas in the later stages the activation of this receptor may sustain tissue damage within the inflamed CNS [42]. Liu et al. observed a limitation of disease progression, driven by a reduced T cell proliferation, CNS infiltration, and cytokine production, in EAE mice treated with an ADORA2a agonist [67]. Tsutsui et al. demonstrated that treatment of EAE mice with caffeine increased the expression of ADORA1 on microglia, with a following reduction of EAE severity. Such effect was further enhanced by a concomitant treatment with the ADORA1 agonist, thus suggesting that ADORA1 represents a therapeutic target to modulate neuroinflammation in MS and other demyelinating diseases [43].

A therapeutic activity of ADO receptor agonists was also observed in the context of other preclinical models of autoimmune diseases. In diabetic mice, a nonselective ADO receptor agonist prevented diabetes development in mice treated with MLDS or cyclophosphamide. Such effect was reverted using ADORA2b but not ADORA1 or ADORA2a antagonists, thus suggesting that only ADORA2b represents a promising therapeutic target for type 1 diabetes [68]. ADORA2a represents a promising therapeutic target for myasthenia gravis. Indeed, it has been demonstrated in rats with experimental autoimmune myasthenia gravis (EAMG) that the stimulation of ADORA2a with a specific agonist reduced the proliferation of acetylcholine receptor- (AChR-) specific autoreactive lymphocytes and the production of anti-AChR antibodies and ameliorated disease severity [69].

In mice with EAU, treatment with CF-101, an agonist of ADORA3, ameliorated pathological manifestation of the disease, through the inhibition of autoreactive T cell proliferation and release of proinflammatory cytokines. This study pointed out a role for ADORA3 as a therapeutic target for human uveitis [70]. In contrast, ADORA2b activation using a specific agonist enhanced the development of the disease in the same preclinical model, through the activation of Th-17 response mediated by dendritic cells [71].

Limited clinical trials are ongoing targeting ADO, ADO receptors, and other related molecules in patients with autoimmune/inflammatory diseases. CF-101 has been tested in phase I, phase IIa, and phase IIb human studies. The treatment was safe and well tolerated and induced an anti-inflammatory and antirheumatic effect in RA patients. Moreover, a significant correlation between ADORA3 expression level and response to drug was reported, thus suggesting that ADORA3 may represent a biologic marker to predict a response to the drug in patients [66, 72, 73].

A phase I/II randomized study was carried out on patients who underwent hematopoietic stem cell transplantation treated with pentostatin, a specific inhibitor of ADA. The results demonstrated that GvHD rates are significantly decreased in patients treated with pentostatin associated with the standard treatment. Accordingly, transplant success rates were higher in treated patients than in controls [74]. A phase II study on HSC-transplanted patients demonstrated a response rate to pentostatin (administered with corticosteroids) of 55% and a survival rate of 78% at 1-2 years [75]. Similarly, a phase II study in children demonstrated a response rate to pentostatin of 53% and a survival rate of 69% at 3 years [76]. In contrast, another phase II trial demonstrated a complete response to pentostatin (administered with corticosteroids) only in 38% of patients and a 9-month overall survival of 47%, with an incidence of severe infections of 57% [77].

## 6. Conclusions

In the last years, several authors reported that extracellular ADO, produced by ectoenzymes belonging to the “classical” adenosinergic pathway (CD39 and CD73), might be involved in the control of the immune response in the context of human autoimmune/inflammatory diseases. Indeed, alterations of the expression and function of both CD39 and CD73 have been reported to be related to the onset of these diseases. On the contrary, overexpression of these molecules, as well as agonists of ADO receptors, is able to mitigate the immune responses and the inflammatory reaction that are crucial for the development of the disease. So far, little is known regarding the expression and function of ectoenzymes that form the “alternative” pathway for ADO production (CD38, CD203a/PC-1, and CD73). We have demonstrated that CD203a/PC-1 is specifically expressed by CD16^−^CD56^bright^ NK cells (that exert regulatory activities) and not by cytotoxic CD16^+^CD56^dim^ NK cells. Moreover, we have demonstrated that the regulatory activity of the former cells is altered in JIA patients, and such alteration was related to a lower expression of CD38 and CD73, thus suggesting that also the “alternative” pathway for ADO production might be altered in autoimmune/inflammatory diseases. In the future, it would be interesting to characterize the expression and function of CD38 and CD203a/PC-1 in other human autoimmune diseases. Indeed, these molecules, as well as CD39 and CD73, might represent a promising target for novel and effective therapeutic approaches.

## Figures and Tables

**Table 1:** Scheme of principal findings regarding the role of ADO and adenosinergic ectoenzymes in autoimmune diseases.

Disease	Molecule(s) involved	Principal finding
Multiple sclerosis	CD73, CD39	Upregulation of CD73 following IFN-*β* treatment (14, 15)
		Loss of regulatory function of CD39^+^ Tregs (16)

EAE	CD73, CD39	Increase in CD73 expression during disease progression (17)
		Resistance to EAE in CD73 KO mice (18, 19)
		Increased enzymatic activity of CD39 and CD73 in EAE mice (20)

CIA	CD73	Upregulation of CD73 and ADORA2A on cells from synovial fluid (21)
		Increased susceptibility to CIA in CD73 KO mice (22)

Rheumatoid arthritis	CD39	Increased frequency of CD39^+^ Treg in PB of patients (23)
		Increased frequency of CD39^+^ Treg in PB of patients responsive to MTX (24)

JIA	CD38, CD73	Decreased expression and function of CD38 and CD73 on synovial CD56^bright^CD16^−^ NK cells from patients (6)
		Lower expression of CD73 in cells from synovial fluid (25)

Diabetes	CD39	Expression of CD39 is related to development of disease in mice (26)
		Lack of ADO receptors A2A and A2B increase susceptibility to the disease (27, 28)

Uveitis	CD73	CD73 mediates the therapeutic effect of MSC in experimental uveitis (31)
		Lack of CD73 expression on *γδ* T cells correlated to high Th17 response (32)
